# Bioactive silicon nitride: A new therapeutic material for osteoarthropathy

**DOI:** 10.1038/srep44848

**Published:** 2017-03-22

**Authors:** Giuseppe Pezzotti, Elia Marin, Tetsuya Adachi, Alfredo Rondinella, Francesco Boschetto, Wenliang Zhu, Nobuhiko Sugano, Ryan M. Bock, Bryan McEntire, Sonny B. Bal

**Affiliations:** 1 Ceramic Physics Laboratory, Kyoto Institute of Technology, Sakyo-ku, Matsugasaki, 606-8126 Kyoto, Japan; 2 Department of Molecular Cell Physiology, Graduate School of Medical Science, Kyoto Prefectural University of Medicine, Kamigyo-ku, Kyoto 602-8566, Japan; 3 The Center for Advanced Medical Engineering and Informatics, Osaka University, Yam daoka, Suita, 565-0871 Osaka, Japan; 4 Department of Dental Medicine, Graduate School of Medical Science, Kyoto Prefectural University of Medicine, Kamigyo-ku, Kyoto 602-8566, Japan; 5 Department of Medical Engineering for Treatment of Bone and Joint Disorders, Osaka University, 2-2 Yamadaoka, Suita, Osaka 565-0854, Japan; 6 Amedica Corporation, 1885 West 2100 South, Salt Lake City, UT 84119, United States; 7 Department of Orthopaedic Surgery, University of Missouri, Columbia, MO 65212, United States.

## Abstract

While the reciprocity between bioceramics and living cells is complex, it is principally governed by the implant’s surface chemistry. Consequently, a deeper understanding of the chemical interactions of bioceramics with living tissue could ultimately lead to new therapeutic strategies. However, the physical and chemical principles that govern these interactions remain unclear. The intricacies of this biological synergy are explored within this paper by examining the peculiar surface chemistry of a relatively new bioceramic, silicon nitride (Si_3_N_4_). Building upon prior research, this paper aims at obtaining new insights into the biological interactions between Si_3_N_4_ and living cells, as a consequence of the off-stoichiometric chemical nature of its surface at the nanometer scale. We show here yet unveiled details of surface chemistry and, based on these new data, formulate a model on how, ultimately, Si_3_N_4_ influences cellular signal transduction functions and differentiation mechanisms. In other words, we interpret its reciprocity with living cells in chemical terms. These new findings suggest that Si_3_N_4_ might provide unique new medicinal therapies and effective remedies for various bone or joint maladies and diseases.

Bioceramics have a long and valued history in orthopaedics^1^. Traditionally, alumina, zirconia, or composites of these compounds were selected because of their bioinertness^2^ while others (*e.g.*, hydroxyapatite or bioglass) were chosen because they possessed extraordinary bioactive surfaces^3^,^4^. Yet, it is now known that all bioceramics profoundly interact with living tissue at the molecular level, even those previously considered to be wholly bioinert^5^. On the one hand, their interactions can be detrimental, leading to destabilization of the ceramic, loss of prosthetic function, and reduced lifetime^6^. On the other hand, they may enhance performance, increase implant lifetime, or even provide protective benefits^5^,^6^. Certain bioceramics can either release or scavenge molecules that support metabolic chemistry, and, most significantly, stimulate cells to replicate and function with exceptional efficiency^7^. We have previously reported about the promising *in vitro* performance of Si_3_N_4_ bioceramics with emphasis on its reciprocity with living cells^8^,^9^,^10^.

The bioactivity of silicate glasses^4^ and the importance of elemental silicon in bone development^11^ have been readily demonstrated in studies over the past 40 years. Silicon assists with the synthesis of glycosaminoglycans and proteoglycans^12^, and it is directly incorporated into hydroxyapatite (HAp) by ionic substitution^13^. Silicic acid (H_4_SiO_4_), the dissolution product of bioactive glasses, was found to be effective in regulating the expression of osteoblastic markers and cell cycle genes^14^. Silicon-substituted hydroxyapatites, porous silicon, and silicon/silica nanoparticles were developed, all sharing the common mechanism of Si-enhanced bioactivity^15^,^16^,^17^. As a complementary element, nitrogen from amino acids is essential for protein synthesis, bone growth, and tissue repair^18^. Cells metabolically assimilate and transform water, carbon dioxide (CO_2_), and three inorganic nitrogen compounds – ammonium (NH_4_^+^), nitrate (NO_3_^−^), and di-nitrogen (N_2_) – into complex biomolecules^19^. One of the more obvious and yet intriguing aspects involving the use of silicon nitride as a biomaterial is that H_4_SiO_4_, NH_4_^+^, NO_3_^−^, and N_2_ are all promptly and copiously available during biological interactions^20^. The peculiar chemistry of Si_3_N_4_, which can be chemically-modulated^21^, facilitates a range of favorable metabolic interactions between both eukaryotic and prokaryotic cells at its inorganic surface^8^,^20^.

It was recently discovered that a post-sintering annealing cycle in nitrogen resulted in an atypical surface modification of Si_3_N_4_ with the formation of peculiar Si-Y-Al-O-N phases. A mixture of these phases, comprehensive of N-apatite and different silicates, appeared as whitish areas in back-scattered scanning electron micrographs. Upon exposure to SaOS-2 cells, the greatest formation of hydroxyapatite coincided with the presence of Si-Y-Al-O-N phases^9^. High cell proliferation and differentiation were confirmed from large concentrations of IGF-1, while corresponding sRANKL assessments showed a very low propensity to form osteoclasts^4^. The average composition of the Si-Y-Al-O-N phases were enriched with yttria (Y_2_O_3_, *i.e.*, a sintering aid which was added along with alumina, Al_2_O_3_, to the raw Si_3_N_4_ powder), and contained N-N bonds, nitrogen dangling bonds, and nitrogen vacancies. Accordingly, Si^4+^ and N^3−^ sites were partially replaced by Y^3+^ and O^2−^, respectively. In the immediate sub-surface, the pristine hexagonal structure of *ß*-Si_3_N_4_ survived, although it had an altered chemical formula, Si_3-3(*m*+*n*)/4_Al_*m*_Y_*n*_O_*x*_N_4-2*x*/3_, with average *m, n*, and *x* values equal to 0.051, 0.021, and 0.166, respectively^21^.

In this study, using advanced surface analyses, the SaOS-2 experiments were repeated on polished N_2_-annealed Si_3_N_4_ samples as a function of time (0–7 days) for the express purpose of obtaining, both *in situ* and *ex situ*, further details on the chemical interactions between cells and the inorganic surface. The full details of the experimental procedures are given in the Supplementary Information.

Figure 1(a) provides a laser-scanning microscopic image of an N_2_-annealed Si_3_N_4_ sample (after interacting with SaOS-2 cells for 3 days). It showed a large number of areas with pronounced hydroxyapatite formation, which preferentially occurred at locations corresponding to the mixture of Si-Y-Al-O-N phases. While this observation confirmed previous findings^8^,^9^, it was newly revealed that there were surface depressions in the vicinity of the apatite deposits. We have interpreted this finding as evidence that the SaOS-2 cells actually *scavenged* molecules from the Si-Y-Al-O-N phases and apparently consumed them to produce bony apatite. More evidence on this speculation will be given later in this paper. Areas below the average planar surface of the sample have been highlighted in Fig. 1(b) for improved visualization. Over the entire surface, it was found that the gain in hydroxyapatite averaged ~167%, which corresponded to the SiYAlON loss. A typical line scan profile is shown in Fig. 1(c), which corresponded to the linear abscissa, *x*, of Fig. 1(a).

The formation of bony apatite was also monitored as a function of time by using photometric optical density analyses of an Alizarin red stain (Fig. 2(a)). Exponential growth of hydroxyapatite appeared to proceed with statistically meaningful repeatability. The trend for time-lapse analyses was also confirmed by *in situ* Raman spectroscopy of the SaOS-2 cellular metabolism as shown in Fig. 2(b) (*cf*., HAp O-P-O symmetric stretching band at ~960 cm^−1^). It also revealed additional HAp structural details including the presence of (CO_3_)^2-^ clusters at the initial growth stage (*cf*. band at ~1100 cm^−1^). As compared to a nearly constant signal from symmetric-ring-breathing in phenylalanine (Phe at ~1005 cm^−1^), a significant decrease in the concentration of nucleic acids was noted at the early stages of cell exposure (*cf*. DNA/RNA bands in the spectral region 780~880 cm^−1^). The observed high concentration of RNA in undifferentiated cells is reportedly similar to high concentrations of non-translated RNA in their early stages of embryogenesis^22^,^23^. With progressing embryogenesis, the non-translated RNA is reactivated via polyadenylyzation in order to synthesize specific proteins required for cell differentiation. Therefore, the smaller intensity for the RNA Raman peak reflects a high degree of cell differentiation. It resulted from RNA translation to produce the specific proteins needed for differentiation. From the Raman spectrum of Fig. 2(b), a nearly constant emission from Cytochrome c was also observed, suggesting that there was no disruption of mitochondrial metabolism or apoptosis^24^,^25^,^26^,^27^.

In the present study, nitric oxide (NO) production within SaOS-2 cells was analyzed as a function of time using diaminofluorescein-2 diacetate and DAF-2 as NO fluorescent probes^28^,^29^. Figure 3 shows optical and fluorescence microscopy images of SaOS-2 cells on an N_2_-annealed Si_3_N_4_ sample for exposure times from zero to 24 h. The sequence of fluorescence images clearly shows an increase in the concentration of NO with increasing exposure to the ceramic substrate. Advanced notions of bone biology have pointed to NO as a key compound in bone remodeling and the pathogenesis of osteoarthritis. The role of nitrogen activity and specifically its effects on osteogenesis were recently reviewed by Saura *et al*.^30^. NO has been identified as a regulator of cellular metabolism, differentiation, and proliferation within the dynamic cascade of events responsible for bone formation and resorption^31^. The ultimate effects of NO strongly depend on its origin, concentration, and microenvironment. It has been reported that the slow release of NO stimulates osteoblast proliferation and differentiation *in vitro*^32^,^33^. *In vivo*, NO is directly produced at low concentrations by osteoblasts and it is responsible for regulating the function of both osteoblasts and osteoclasts^31^. However, at high concentrations, NO is a potent inhibitor of osteoclast-mediated bone resorption. In combination with other cytokines, IFN-γ markedly induces NO production, involving suppression of both osteoclast formation and the activity of mature osteoclasts^34^. When seen in the light of these prior reports, the results of Fig. 3 strongly demonstrate the role in osteogenesis of the nitrogen scavenged from the substrate and the link between NO activity and the enhanced proliferation of the SaOS-2 cells.

Figures 4, 5, 6, and show a series of backscattered SEM micrographs of the surface of a N_2_-annealed Si_3_N_4_ sample after increasing exposure to SaOS-2 cells (*i.e.*, 0, 3, and 7 days, respectively). In Fig. 4(a), the mixture of Si-Y-Al-O-N phases is the brighter “icing-like” area within the photograph. This ~100 nm thick phase formed during the post-sintering annealing cycle in N_2_ gas. It is a crystalline surface byproduct of the intergranular glassy phase present in sintered Si_3_N_4_^21^. A comparison between normalized cathodoluminescence (CL) spectra collected at Si-Y-Al-O-N and Si_3_N_4_ sites is given in Fig. 4(b) (*cf*. locations A and B, respectively, in the micrograph (a)). CL spectroscopy, which is quite sensitive to the presence of lattice defective sites, clearly revealed a common off-stoichiometric state in both the investigated areas of the material’s surface (*i.e*., the CL probe likely reached the substrate also at location A). However, clear differences could be detected by comparing spectra from the two locations. A N-vacancy-related band, V_N_^3+^, at around 500 nm was the strongest emission for both locations A and B, which indicates the presence of a large concentration of N-vacancies with a 3^+^ charge. Substitutions of Al or Y on Si sites in the Si-Y-Al-O-N mixture also resulted in a significant increase of positively charged N-N bonds, N_4_^+^, and negatively charged N-dangling bonds, N_2_^0^. These lattice defects were stronger at location A (*i.e.*, the area covered by the Si-Y-Al-O-N phases) and correlated to locations where the SaOS-2 cells preferentially clustered^9^. A striking feature in the CL spectrum of the Si-Y-Al-O-N phases was the appearance of three extremely sharp sub-bands located at 564, 632, and 745 nm (labeled with an asterisk in Fig. 4(b)). These latter three bands are part of a quadruplet (*i.e*., an additional band is located at around 604 nm but it is hidden by the V_N_^3+^ emission) and belong to the emission activity of Si quantum dots (Si-QDs)^35^,^36^,^37^. X-ray photoelectron spectroscopy (XPS) revealed a triplet corresponding to Si-Si, Si-N, and Si-O bonds at increasing binding energies (99.3, 101.3, and 103.1 eV, respectively), which confirmed the presence of Si-QDs (and silicon oxide).

The surface morphological details of an N_2_-annealed Si_3_N_4_ sample after 3-days exposure to SaOS-2 cells are depicted in Fig. 5 along with the CL analyses. CL spectra were collected at two distinct zones: (i) a protrusion where large hydroxyapatite osteocytes were formed (location A), and, (ii) a relatively flat area previously covered by cells but at the early stage of apatite formation (location B). The CL spectra from these two zones are provided in separate insets to Fig. 5. Although they possessed some common features, their respective CL spectra differed markedly. The large osteocyte agglomeration at location A displayed a CL emission consisting of five sub-bands labeled as (I)~(V) assigned to: a Si-doped hydroxyapatite near-bandgap emission (weak sub-band (I) at ~345 nm)^38^, defective clusters, O = C-O_2_, at carbonate sites in hydroxyapatite ((II) at ~402 nm)^39^, oxygen vacancies, V_O_^+^ and V_O_, in hydroxyapatite ((III) and (IV) at ~506 and ~620 nm) most likely created by the introduction of Si in the hydroxyapatite lattice^40^, and a weaker band at around 710 nm, which is associated with a V_O_ emission from a shallow donor ((V) labeled SD). The CL spectrum retrieved at the flat area of location B, possessing incipient but yet unsubstantial hydroxyapatite formation, was also deconvoluted into a number of sub-bands. For this location, the CL probe revealed the main stoichiometric defects of the substrate (*cf.,* inset to Fig. 5 with Fig. 1). However, the oxygen-related defective bands labeled as III (oxygen vacancies in hydroxyapatite) and IV (oxygen excess-sites in silica) were also clearly detected. Nevertheless, the most striking feature in the CL spectrum of location B was the strong and sharp quadruplet emission arising from quantum confinement in Si-QDs.

The experiments were repeated on samples exposed to SaOs-2 cells for 7 days. These samples were all conspicuously covered by hydroxyapatite. A small portion of the hydroxyapatite layer was mechanically detached on one sample in order to observe the buried surface (Fig. 6(a)). Underneath the hydroxyapatite were copious amounts of Si-Y-Al-O-N phases, similar to the ones shown in Fig. 4(a). Different locations on the thick apatite layer (location A) and the buried zone (location B) were subsequently examined by Raman spectroscopy. The Raman spectrum in Fig. 6(b), collected at location A, showed strong hydroxyapatite and phenylalanine bands, while the band at ~1100 cm^−1^ related to (CO_3_)^2−^ clusters evidently disappeared. In stoichiometric bony apatite the P-O stretching Raman band is a singlet and appears at around 960 cm^−1^. The Raman band detected at location A clearly consisted of two sub-bands and showed significant asymmetry. This latter characteristic arose from the presence of a sub-band located at ~946 cm^−1^ (labeled *A*_*1*_ in Fig. 6(b)) in addition to the main component at ~960 cm^−1^ (labeled *A*_*2*_). The physical meaning of the low-frequency sub-band *A*_*1*_ can be discussed according to fundamental crystallographic data presented by Zou *et al*.^41^. Those researchers examined the enhanced bioactivity of silicon-doped apatites by comparing XRD and Raman spectroscopy data. They found that, when silicon was incorporated into the apatite lattice structure to form (SiO_4_)^4−^ tetrahedra, the Raman spectrum at around 960 cm^−1^ showed clear asymmetry and degenerated into a doublet with a low-frequency component. The bony apatite deposited by the SaOS-2 cells in the current study showed this same chemical fingerprint. Energy dispersive X-ray (EDX) analyses at location A revealed a Ca-deficient structure (Ca/P at. ratio equal to 1.55 ± 0.55). EDX analyses at thick hydroxyapatite locations also substantiated the presence of trace Si (0.11 ± 0.06 at.%).

An additional examination of the bony apatite deposited by SaOS-2 cells was performed by FT-IR spectroscopy. Figure 6(c) shows the FT-IR spectrum of the sample exposed for 7 days to SaOS-2 cells. This complex and overlapping spectrum was deconvoluted and labeled according to literature data^42^,^43^,^44^,^45^,^46^,^47^. Contributions from hydroxyapatite (PO_4_)^3−^, proteins, and collagen bonds (labeled as “organic” in Fig. 6) appeared in the frequency range between 900 and 1000 cm^−1^. A relatively strong emission from tricalcium phosphate (α- and β-TCP) appeared at ~875 cm^−1^, which belonged to O-C-O bending in (CO_3_)^48^, was found neighboring bands from Si-O-Si bonds, proteins, and Si-OH bonds. Except for this latter TCP emission, the zone between 875 and 900 cm^−1^ is largely silent for TCP and hydroxyapatite phases. This vibrational zone provided the clearest fingerprint for the modified chemistry of the bony apatite produced by SaOS-2 cells. It showed a small but clearly detectable shoulder at 893 cm^−1^ belonging to the triply degenerate asymmetric stretching of the (SiO_4_)^4−^ tetrahedra^45^. An additional band at ~932 cm^−1^ was also related to the (SiO_4_)^4−^ tetrahedra, but it overlapped with Si-OH bond vibrations (also present at 840 cm^−1^)^45^.

The CL spectra from location B after 7 days of exposure to the SaOS-2 cells (Fig. 6(d)) was in agreement with that collected at location A in Fig. 4(a) (i.e., prior to exposure to the SaOS-2 cells). On the other hand, the CL spectrum collected at location B in Fig. 6 showed stronger emissions from Si-QDs as compared to that retrieved at location A of Fig. 4, which is probably due to a locally higher thickness of the Si-Y-Al-O-N phase mixture. The spectrum emitted from Si-QDs was obtained by subtracting the spectrum of A from that in B, as shown in Fig. 6(d).

By using different analytical techniques, the resulting data clearly indicate that biochemical reactions occurring at the Si_3_N_4_ surface provided silicic acid (H_4_SiO_4_) and ammonia (NH_3_), both of which were active in regulating cellular metabolism. It is postulated that osteoblast cells actually scavenged Si from silicic acid and used part of it to build (SiO_4_)^4−^ tetrahedra while endocytotically internalizing Si-QDs. In turn, the newly formed Si-apatite and the Si-QDs affected the morphogenetic activity of the osteoblasts through expressing osteoprotegerin (OPG) and the bone morphogenetic protein 2 (BMP2); and while strongly stimulating osteoblasts, they also inhibited formation of osteoclasts^49^. The current findings explain previous sRANKL and IGF-1 assessments^9^, as well as the Alizarin red stain optical density analyses (Fig. 2(a)). These CL spectroscopic experiments suggest that: (i) the preferential substrate location of the SaOS-2 cells was on the Si-Y-Al-O-N compounds rich in Si-QDs (*cf.*, bands labeled “*” in Fig. 5 and the XPS analyses of the Si bonds); (ii) the Si-QDs stimulated the formation of osteoblasts; and, (iii) part of the scavenged Si entered into the newly formed Ca-defective hydroxyapatite lattice (*cf.*, Bands (III)~(V) in Fig. 5, EDX analyses, Raman and FT-IR analyses in Fig. 6). Additionally, the metabolic maintenance of the SaOS-2 cells depended upon their ability to efficiently extract nitrogen from the Si-Y-Al-O-N phases for use as a source of energy and for protein formation. It is speculated that nitrogen scavenging occurred through NH_4_^+^ uptake driven by the membrane potential. Previously, pH microscopy experiments demonstrated that ammonia formation and pH buffering readily occurs at the surface of Si_3_N_4_ when exposed to a biological environment^20^. Surface alkalization might also play a role in enhancing the metabolic uptake of nitrogen^50^. Finally, the stable Cytochrome c metabolism observed during the *in situ* Raman monitoring of Fig. 2(b) demonstrates that, unlike cadmium telluride, alumina, titania, and silver nanoparticles^24^,^25^,^26^,^27^, the Si-QDs in Si-Y-Al-O-N compounds do not decrease the expression or impair the mitochondrial functions of the SaOS-2 cells. The postulated metabolic activity of these osteosarcoma cells on the surface of N_2_-annealed Si_3_N_4_ is illustrated in Fig. 7(a), while Fig. 7(b) schematically shows how the SaOS-2 cells preferentially locate on Si-QDs, generate carbonated hydroxyapatite, then copiously deposit Ca-deficient hydroxyapatite while incorporating (SiO_4_)^4−^ tetrahedra in the deposited apatite, and perhaps endocytotically internalize the Si-QDs.

This study identified a number of peculiar chemical agents (*i.e*., H_4_SiO_4_, NH_4_^+^, and Si-QDs) affecting the metabolic activity of living SaOS-2 cells while residing on the surface of biomedical Si_3_N_4_. Having observed these physicochemical interactions, the next logical question is: What functional role(s) can they beneficially serve within the human body? In answering this question, one needs to look no further than finding countermeasures to chronic bone diseases such as rheumatoid arthritis^51^, osteolysis^52^, bacterial infections such as periodontitis^53^, and estrogen deficiency^54^. The present findings may have important implications in counteracting some of these bone maladies. It should be noted that Si_3_N_4_ spinal implants are nowadays successfully employed and show excellent osteogenesis^55^. Moreover, pH microscopy data collected during a bacterial study on the surface of Si_3_N_4_ revealed pH buffering effects due to surface reactions involving charged nitrogen compounds^20^. These properties could be exploited in local treatments of osteoporotic bone segments (i.e., vertebral body) through a local implantation. Moreover, the pH buffering effect could attenuate inflammation and infections. The present data triggers future studies to address whether or not Si-QDs-containing Si-Y-Al-O-N phases at the surface of Si_3_N_4_ bioceramics, in contact with diseased bone, could locally quench osteoclastic resorption activities, while concurrently promoting further structural remodeling via osteoblasts. As a “solid-state medicine,” engineered Si_3_N_4_ surfaces including Si-Y-Al-O-N compounds with improved bone healing capacity could be applied to the clinical practice. In some cases, they could even replace bisphosphonates or other anti-resorptive drugs^56^, leading to the local regulation and differentiation of osteoclasts and their apoptosis. As another example, osteolysis (which results from polyethylene wear debris in artificial hip joints) could be prevented, counteracted, or even healed by “glazing” a cementless metallic stem with a Si_3_N_4_ phase of suitable stoichiometry, or by introducing this phase as filler in bone cement. The Si-Y-Al-O-N phases and the Si-QDs contained in it might boost the osteogenic properties of Si_3_N_4_ bioceramics by further regulating the local bone microenvironment without affecting osteoclastic activity in areas such as lymphoid and other tissues with immune-related functions. While researchers have focused on developing stronger and more bioinert materials, designing a new generation of “smart” materials that metabolically interact with cells may lead to the prevention or cure of osteolysis and other bone diseases.

## Methods

SaOS-2 human osteosarcoma cells were first cultured and incubated in 4.5 g/L glucose DMEM (D-glucose, L-Glutamine, Phenol Red, and Sodium Pyruvate) supplemented with 10% fetal bovine serum. They were allowed to proliferate within petri dishes for about 24 h at 37 °C. The final SaOS-2 concentration was 5 × 105 cell/ml. The cultured cells were then deposited on the top surface of N_2_ heat-treated Si_3_N_4_ disks previously sterilized by exposure to UV light. In the osteoconductivity tests, cell seeding took place in an osteogenic medium, which consisted of DMEM supplemented with about 50 μg/mL ascorbic acid, about 10 mM β-glycerol phosphate, 100 mM hydrocortisone, and about 10% fetal bovine calf serum. The samples were incubated up to 7 days at 37 °C. The medium was changed twice during the incubation period.

Bone mineralization was determined by Alizarin Red S (Sigma Aldrich) upon staining at 7 days. Cells were washed twice and then fixed in 95% ethanol for 10 min at room temperature. Following washing in distilled water, cells were stained with 40 mM Alizarin Red S for 10 min at room temperature. For the quantification of staining density, concentrations were determined by measuring the absorbance at 562 nm.

The results were statistically analyzed using the unpaired Student’s t-test or by one-way Analysis of Variance (ANOVA). A value of p < 0.05 was considered significant and labeled with one asterisk, while a p value <0.003 was labeled with two asterisks.

Real-time detection of nitric oxide (NO) production in living cells was obtained through fluorescence imaging by means of a membrane permeable fluorescent indicator DAF-2(NO) (Goryo Chemical, Inc., Sapporo, Japan). This indicator consisted of diaminofluorescein-2 diacetate. Once inside the SaOS-2 cells, this substance is deacetylated by intracellular esterizes and can be detected with excitation/emission maxima of 495/515 nm. The dye-loaded cells were incubated at 37 °C osteogenic medium (complete medium supplemented with ≫50 μg/mL ascorbic acid, 10 mM β-glycerol phosphate, and 100 nM dexamethasone) and observed *in situ* on the ceramic substrate at increasing exposure times between 0 and 24 h at intervals of 8 h. Fluorescent intensities of DAF-2 were determined by confocal microscopy (BZ X710, Keyence, Japan). Dye-loaded cells were excited with the 488 nm of a Krypton/Argon laser for DAF-2, and increases in DAF-2 fluorescence were monitored *in situ* with the SaOS-2 cells on the ceramic substrate.

*In situ* Raman microscopy images were collected on living SaOS-2 cells using a dedicated instrument (RAMANtouch, Nanophoton Co., Osaka, Japan) with a 20x immersion-type optical lens. This spectroscope allowed ultra-fast imaging of up to 400 spectra simultaneously, thus collecting average spectra in a time faster than the movement of cells. The excitation source was at 785 nm and the spectral resolution was 1.2 cm^−1^ (spectral pixel resolution equal to 0.3 cm^−1^/pixel).

Fourier Transform Infrared Spectroscopy (FT-IR) was carried out using the imaging system Spotlight 200 (Perkin Elmer, Waltham, Massachusetts, USA) equipped with an attenuated total reflectance (ATR) imaging attachment. FT-IR spectra were acquired at aperture size of 200 × 200 μm^2^.

Scanning electron microscopy (SEM) was carried out on the N_2_-annealed Si_3_N_4_ surface using a scanning electron microscope (JSM-6010LA, JEOL Ltd.). Micrographs were collected in a mix mode including backscattered electrons. The microscope was equipped with an energy dispersive X-ray spectroscopy (EDS) device for elemental mapping. All samples were sputter-coated (108auto, Cressington, Watford, UK) with a thin (~20–30 Å) layer of gold. Samples were imaged using an accelerating voltage of 10 kV at working distances of 7~10 mm and spot sizes of 4~4.5 mm. Laser-scanning micrographs of the sample surfaces after exposure to SaOS-2 cells were collected by means of a 3D laser-scanning microscope (VK-X200 K Series, Keyence, Osaka, Japan) using a 150x objective lens, with a numerical aperture of 0.9.

Cathodoluminescence (CL) spectra were collected in a field-emission gun scanning electron microscope (FEG-SEM, SE-4300, Hitachi Co., Tokyo, Japan). For all experiments, the same experimental conditions were applied (acceleration voltage and beam current fixed at 5 kV and 180 pA, respectively). The electron-stimulated luminescence was analyzed by a high spectrally resolved monochromator (Triax 320, Jobin-Yvon, Horiba Group, Tokyo, Japan). Spectral deconvolution into Lorentzian bands was made by means of commercially available software (Origin 9.1, OriginLab Co., Northampton, MA, USA).

X-ray photoelectron spectroscopy (XPS) experiments of the Si 2p photoelectron spectra were conducted in a JEOL JSP-9010MC/SP device with an MgKa source at an angle of 34°. Output, pass energy, voltage step, and dwell time were 10.0 kV x10.0 mA, 10 eV, 0.1 eV, and 100 ms, respectively.

## Additional Information

**How to cite this article:** Pezzotti, G. *et al*. Bioactive silicon nitride: A new therapeutic material for osteoarthropathy. *Sci. Rep.* **7**, 44848; doi: 10.1038/srep44848 (2017).

**Publisher's note:** Springer Nature remains neutral with regard to jurisdictional claims in published maps and institutional affiliations.

## Supplementary Material

Supplementary Information

## Figures and Tables

**Figure 1:**
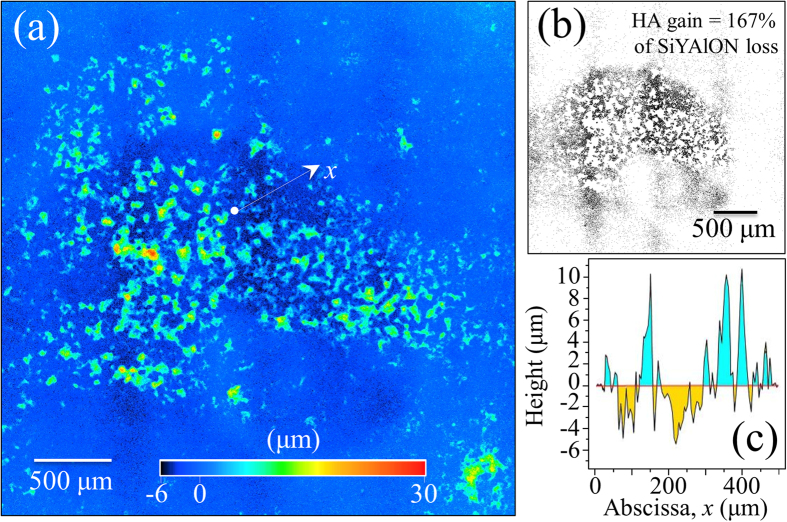
(**a**) Laser-scanning microscopic image of the N_2_-annealed Si_3_N_4_ sample after osteoconductivity test. The protrusions correspond to sites of apatite formation; (**b**) the zones laying below the average zero level in the same area in (**a**) are highlighted with black contrast for better visualization; and, (**c**) a typical profile line-scan corresponding to the linear abscissa, *x*, drawn in (**a**).

**Figure 2:**
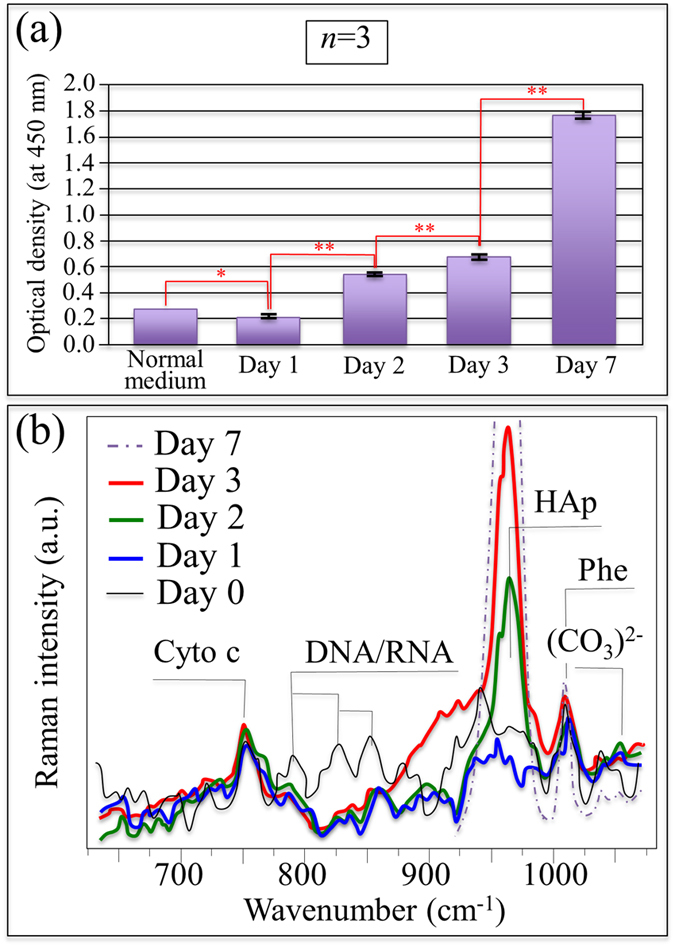
Results of time-dependent optical density of Alizarin red stain (**a**) and *in situ* Raman spectroscopy on living SaOS-2 cells (**b**) exposed to the N_2_-annealed Si_3_N_4_ substrate.

**Figure 3:**
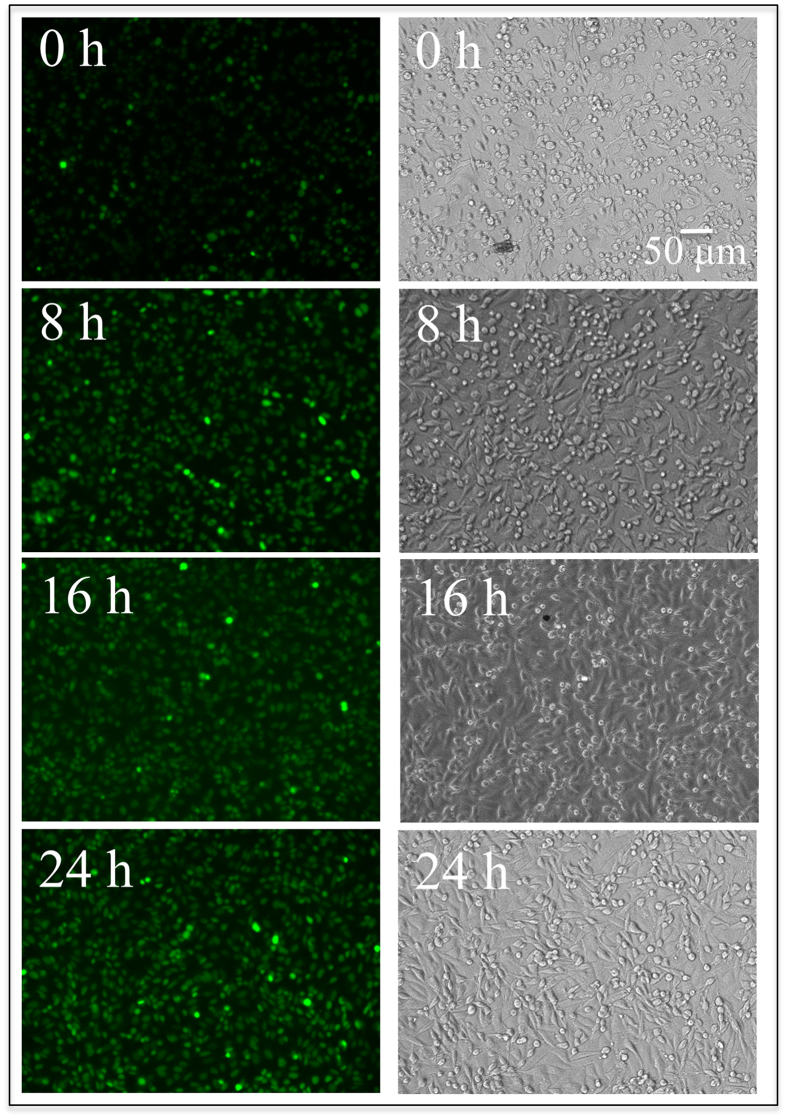
Optical micrographs and fluorescence images of DAF-2 dye-loaded SaOS-2 cells observed *in situ* on the ceramic substrate at increasing exposure times between 0 and 24 h at intervals of 8 h.

**Figure 4:**
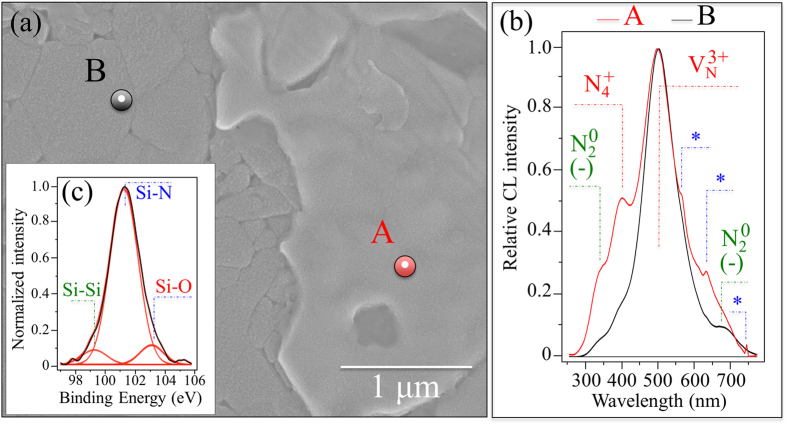
(**a**) Backscattered SEM micrograph of the N_2_-annealed Si_3_N_4_ sample (the brighter area locates Si-Y-Al-O-N phases); in (**b**) a comparison between normalized CL spectra collected at locations A and B in (**a**); and, in (**c**), the XPS Si *2p* photoelectron spectrum revealing the concurrent existence of Si-Si, Si-O, and Si-N bonds.

**Figure 5:**
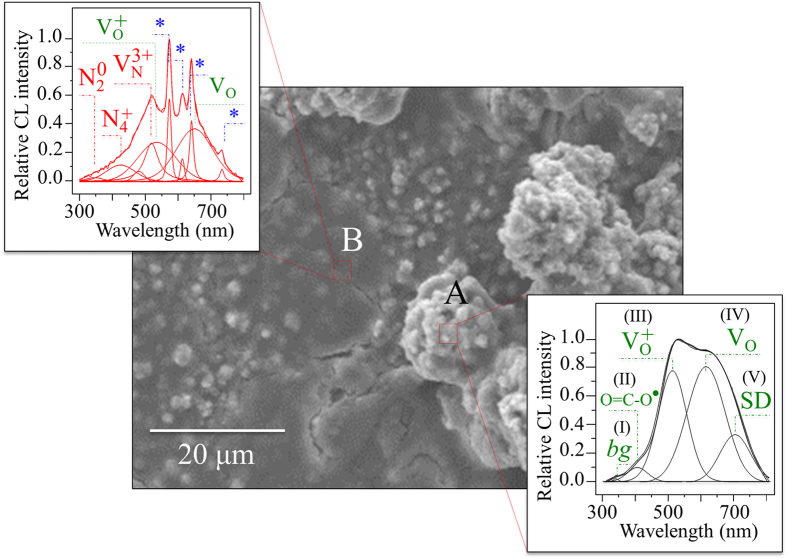
Electron micrograph showing the morphology of the N_2_-annealed Si_3_N_4_ sample after 3 days exposure to SaOS-2 cells. CL spectra were collected, as shown in inset, at a large hydroxyapatite osteocyte (**A**) and a relatively flat zone at the early stage of apatite formation (**B**). Labels of the CL deconvoluted sub-bands are described in the text.

**Figure 6:**
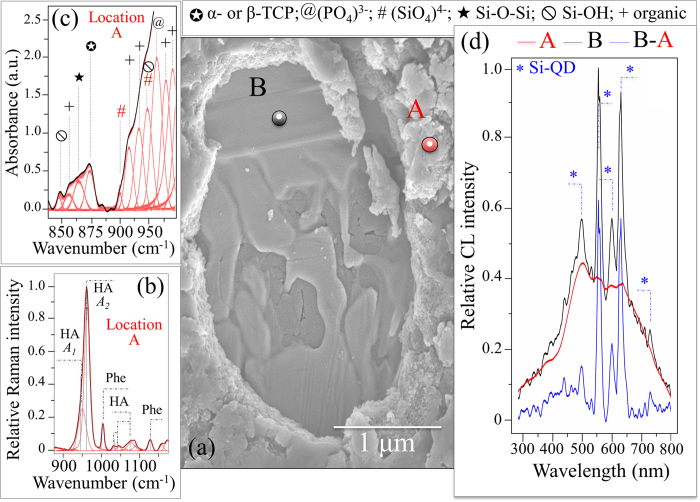
Electron micrograph showing the morphology of the N_2_-annealed Si_3_N_4_ sample after 7 days exposure to SaOS-2 cells. The zone imaged in (**a**) shows a relatively thick layer of hydroxyapatite (*cf*. Raman spectrum in (**b**)) with a zone in which a layer was mechanically removed. In this zone, note the “icing” Si-Y-Al-O-N phases on which the SaOS-2 cells covered the hydroxyapatite layer. In (**c**), the FT-IR spectrum shows the fingerprint for (SiO_4_)^4−^ substitution in bony apatite as described in the text. Subtracting the CL spectrum retrieved at location A (hydroxyapatite layer) from that at location B (Si-Y-Al-O-N) singles out the CL emission spectrum of Si-QDs (**d**).

**Figure 7:**
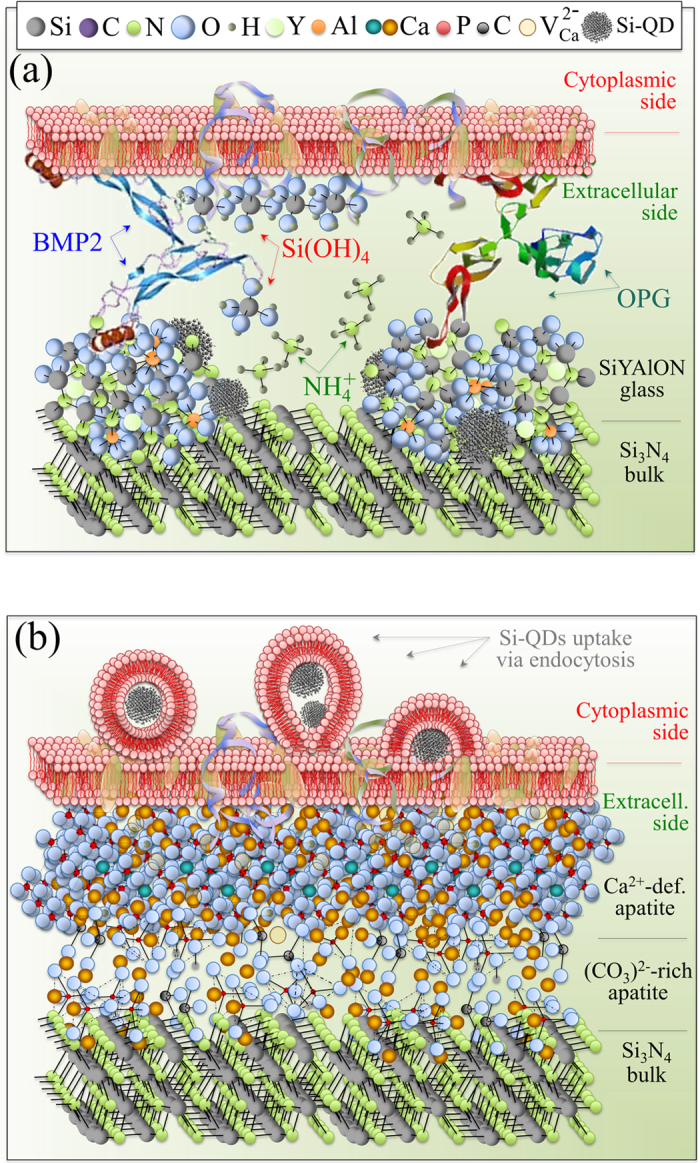
Schematic illustrations of the hypothesized metabolic activity of SaOS-2 cells on the surface of N_2_-annealed Si_3_N_4_: (**a**) early stage interaction with elementary molecules emitted from the Si_3_N_4_ surface and release of OPG and BMP2; and, (**b**) deposition of carbonate apatite and hydroxyapatite layers with concurrent endocytosis of Si-QDs.
