# Supplementary material for: Bioactive silicon nitride: A new therapeutic material for osteoarthropathy
Source: Sci Rep. 2017 Mar 22;7:44848. doi: 10.1038/srep44848 (PMC5361106; doi:10.1038/srep44848)
Supplement: Supplementary Information [file srep44848-s1.doc]

**Bioactive silicon nitride: A new therapeutic material for**

**osteoarthropathy**

Giuseppe Pezzotti^1,2,3*^, Elia Marin^1^, Tetsuya Adachi^4^, Alfredo Rondinella^1^, Francesco Boschetto^1^, Wenliang Zhu^5^, Nobuhiko Sugano^5^, Ryan M. Bock^6^, Bryan McEntire^6^, and Sonny B. Bal^6,7^

*^1^Ceramic Physics Laboratory, Kyoto Institute of Technology,*

*Sakyo-ku, Matsugasaki, 606-8126 Kyoto, Japan*

*^2^Department of Molecular Cell Physiology, Graduate School of Medical Science,*

*Kyoto Prefectural University of Medicine, Kamigyo-ku, Kyoto 602-8566, Japan*

*^3^The Center for Advanced Medical Engineering and Informatics, Osaka University, Yamadaoka, Suita, 565-0871 Osaka, Japan*

*^4^Department of Dental Medicine, Graduate School of Medical Science,*

*Kyoto Prefectural University of Medicine, Kamigyo-ku, Kyoto 602-8566, Japan*

*^5^Department of Medical Engineering for Treatment of Bone and Joint Disorders,*

*Osaka University, 2-2 Yamadaoka, Suita, Osaka 565-0854, Japan*

*^6^Amedica Corporation, 1885 West 2100 South, Salt Lake City, UT 84119*

*^7^Department of Orthopaedic Surgery, University of Missouri, Columbia, MO 65212*

**Supplementary information**

The Si_3_N_4_ samples used in the present study were produced by Amedica Corp., (Salt Lake City, UT), using conventional ceramic fabrication techniques. The material contained minor fractions of Y_2_O_3_ and Al_2_O_3_ as sintering aids. The resulting microstructure exhibited a bimodal granular population, consisting of a minor fraction of relatively large acicular *ß*-Si_3_N_4_ grains embedded in a finer grain matrix. The sintering additives resulted in thin grain boundaries and multiple grain junctions composed of either amorphous or crystalline silicon-yttrium-aluminum-oxynitride (SiYAlON), respectively. Surfaces of as-fired Si_3_N_4_ samples were finely polished and then subjected to nitrogen heat treatment at 1400^o^C for 30 min under 1~2 psi of filtered N_2_ gas. The high-temperature N_2_-atmosphere treatment was employed to maximize the surface density of amine relative to hydroxyl groups and to cover the sample with a peculiar SiYAlON phase. All tested samples were prepared as disks with diameter and thickness equal to 12 mm and 1 mm, respectively. All samples were finely polished before N_2_-annealing and the influence of surface roughness was not considered to be a significant variable within this study.

SaOS-2 human osteosarcoma cells were first cultured and incubated in an osteoblast-inducer medium consisting of 4.5 g/L glucose DMEM (D-glucose, L-Glutamine, Phenol Red, and Sodium Pyruvate) supplemented with 10% fetal bovine serum. They were allowed to proliferate within petri dishes for about 24 h at 37^o^C. The final SaOS-2 concentration was equal to 5 x10^5^ cell/ml. The cultured cells were then deposited on the top surface of N_2_ heat-treated Si_3_N_4_ disks previously sterilized by exposure to UV light. In the osteoconductivity tests, cell seeding took place in an osteogenic medium, which consisted of DMEM supplemented with about 50 *μ*g/mL ascorbic acid, about 10 mM β-glycerol phosphate, 100 mM hydrocortisone, and about 10% fetal bovine calf serum. The samples were incubated up to 7 days at 37^o^C. The medium was changed twice during the incubation period. Mineralization was visualized by fixing cells in 75% ethanol for 30 minutes at 4°C followed by staining with Alizarin Red-S (40 mM) for 10 minutes. Excess stain was removed by copious washing with distilled water. Osteoconduction and staining tests were repeated three times (*n*=3).

Real-time detection of nitric oxide (NO) production in living cells was obtained through fluorescence imaging by means of a membrane permeable fluorescent indicator DAF-2(NO) (Goryo Chemical, Inc., Sapporo, Japan). This indicator consisted of diaminofluorescein-2 diacetate. Once inside the SaOS-2 cells, this substance is deacetylated by intracellular esterases and can be detected with excitation/emission maxima of 495/515 nm. The dye-loaded cells were incubated at 37 ^o^C osteogenic medium (complete medium supplemented with ≈50 μg/mL ascorbic acid, 10 mM β-glycerol phosphate, and 100 nM dexamethasone) and observed *in situ* on the ceramic substrate at increasing exposure times between 0 and 24 h at intervals of 8 h. Experiments using DAF-2 were performed in a dark room, because the dye is light sensitive. Then, the samples were transferred to a chamber on the stage of the fluorescence microscope (BZ X710, Keyence, Japan), and fluorescent intensities of DAF-2 were determined by confocal microscopy. Dye-loaded cells were excited with the 488 nm of a Krypton/Argon laser for DAF-2, and increases in DAF-2 fluorescence were monitored *in situ* with the SaOS-2 cells on the ceramic substrate.

*In situ* Raman microscopy images were collected on living SaOS-2 cells using a dedicated instrument (RAMANtouch, Nanophoton Co., Osaka, Japan) with a 20x immersion-type optical lens. This spectroscope allowed ultra-fast imaging of up to 400 spectra simultaneously, thus collecting average spectra in a time faster than the movement of cells. The excitation source was at 785 nm and the spectral resolution was 1.2 cm^-1^ (spectral pixel resolution equal to 0.3 cm^-1^/pixel).

Fourier Transform Infrared Spectroscopy (FT-IR) was carried out using the imaging system Spotlight 200 (Perkin Elmer, Waltham, Massachusetts, USA) equipped with an attenuated total reflectance (ATR) imaging attachment. FT-IR spectra were acquired at aperture size of 200×200 µm^2^.

Scanning electron microscopy (SEM) was carried out on the N_2_-annealed Si_3_N_4_ surface using a scanning electron microscope (JSM-6010LA, JEOL Ltd.). Micrographs were collected in a mix mode including backscattered electrons. The microscope was equipped with an energy dispersive X-ray spectroscopy (EDS) device for elemental mapping. All samples were sputter-coated (108auto, Cressington, Watford, UK) with a thin (~20-30 Å) layer of gold. Samples were imaged using an accelerating voltage of 10 kV at working distances of 7~10 mm and spot sizes of 4~4.5 mm. Laser-scanning micrographs of the sample surfaces after exposure to SaOS-2 cells were collected by means of a 3D laser-scanning microscope (VK-X200K Series, Keyence, Osaka, Japan) using a 150 objective lens, with a numerical aperture of 0.9.

Cathodoluminescence (CL) spectra were collected in a field-emission gun scanning electron microscope (FEG-SEM, SE-4300, Hitachi Co., Tokyo, Japan). For all experiments, exactly the same experimental conditions were applied (acceleration voltage and beam current fixed at 6 kV and 180 pA, respectively). The electron-stimulated luminescence was analyzed by a high spectrally resolved monochromator (Triax 320, Jobin-Yvon, Horiba Group, Tokyo, Japan). Spectral deconvolution into Lorentzian bands was made by means of commercially available software (Origin 9.1, OriginLab Co., Northampton, MA, USA).

X-ray photoelectron spectroscopy (XPS) experiments of the Si 2*p* photoelectron spectra were conducted in a JEOL JSP-9010MC/SP device with an Mg*K_α_* source at an angle of 34^o^. Output, pass energy, voltage step, and dwell time were 10.0 kV x10.0 mA, 10 eV, 0.1 eV, and 100 ms, respectively.
